# The factors associated with clozapine polypharmacy for schizophrenia patients discharged from a large public psychiatric hospital in Taiwan, 2006–2021

**DOI:** 10.1097/MD.0000000000040897

**Published:** 2024-12-20

**Authors:** Yu-Ju Shih, Ching-Hua Lin, Li-Shiu Chou

**Affiliations:** a Kaohsiung Municipal Kai-Syuan Psychiatric Hospital, Kaohsiung, Taiwan; b Department of Psychiatry, School of Medicine, College of Medicine, Kaohsiung Medical University, Kaohsiung, Taiwan; c Department of Post-Baccalaureate Medicine, College of Medicine, National Sun Yat-Sen University, Kaohsiung, Taiwan.

**Keywords:** clozapine monotherapy, clozapine polypharmacy, schizophrenia, temporal trend

## Abstract

Clozapine treatment continues to be recognized as the gold standard for managing treatment-resistant schizophrenia. Combining clozapine with other antipsychotics (i.e., clozapine polypharmacy) has emerged as an option for clozapine-resistant schizophrenia. We aimed to investigate the factors associated with clozapine polypharmacy in schizophrenia patients discharged on clozapine from a public psychiatric hospital. The analysis included patients with schizophrenia who were discharged between 2006 and 2021 and prescribed clozapine upon discharge. All patients were divided into 2 groups: clozapine monotherapy and clozapine polypharmacy. Multivariate logistic regression was used to identify factors associated with clozapine polypharmacy. A total of 1396 (42.7%) schizophrenia patients discharged on clozapine polypharmacy. In a multivariate logistic regression model, the clozapine polypharmacy was more likely to be male gender, to be younger, to be earlier age of onset, to have a greater number of previous hospitalizations, to have a shorter length of hospital stay, and to have a lower clozapine daily dose. The prevalence of clozapine significantly increased from 22.4% in 2006 to 50% in 2021. Compared with clozapine monotherapy, clozapine polypharmacy was associated with male gender, younger, earlier age of onset, a greater number of previous hospitalizations, shorter length of hospital stay, and lower clozapine daily dose. The utilization of clozapine polypharmacy has seen a significant increase over time. Further research is necessary to clarify its efficacy, safety, and overall risk/benefit ratio.

## 1. Introduction

Clozapine is considered the primary treatment of choice for treatment-resistant schizophrenia (TRS) according to several guidelines.^[1–3]^ The common definition of TRS requires nonresponse to at least 2 or more antipsychotic medications, each for adequate dose (at least 600 mg chlorpromazine equivalent per day), duration (at least 6 weeks) and adherence (at least 80% of the prescribed doses).^[3]^ Approximately 20% to 30% of patients with schizophrenia meet criteria for TRS.^[4,5]^ However, not all patients with TRS respond well to clozapine alone. Besides, it may often be difficult for some patients to tolerate with the side effects of clozapine. Common side effects of clozapine encompass dizziness, hypersalivation, weight gain, tachycardia, and constipation. In addition, there are potentially dangerous side effects, including myocarditis, seizures, agranulocytosis or granulocytopenia, and gastrointestinal hypomotility.^[6–8]^ Therefore, the prescription rates of clozapine are in fact lower than the prevalence of TRS in certain countries. For example, in Denmark, the clozapine prescription rate is 10.2%, while in the United States, it ranges from 2% to 15%.^[9–11]^ Moreover, in Asia, the proportion of patients receiving clozapine prescriptions is only around 14.5% to 15.9%.^[12]^ When confronted with treatment-resistant schizophrenia, particularly in cases where clozapine fails to produce the desired response or is discontinued due to side effects, clinicians have increasingly turned to the strategy of co-prescription clozapine with other antipsychotics (i.e., clozapine polypharmacy).

The benefits of clozapine polypharmacy are limited and controversial.^[13]^ While a meta-analysis study has indicated that augmentation with a second antipsychotic can be modestly beneficial for patients who do not respond fully to clozapine,^[14]^ the risks and side effects associated with clozapine polypharmacy need to be carefully considered. Prescribing additional antipsychotics with clozapine may be associated with an increased risk of extrapyramidal side effects, elevated serum prolactin levels,^[15]^ heightened risk of agranulocytosis^[16,17]^ and QTc prolongation.^[18]^

Nevertheless, it is undeniable that in the actual clinical world, clozapine polypharmacy is common, with around 30% to 50% of individuals were prescribed with a second antipsychotic medication on clozapine.^[19,20]^ However, there is still no consistent evidence regarding which patients should be used for clozapine augmentation, and there have been few studies that have indicated the factors that may be associated with prescription of clozapine polypharmacy. The aim of this study was to acquire knowledge about clozapine antipsychotic augmentation prescribing patterns, investigate the factors associated with clozapine polypharmacy in schizophrenia patients, and analyze the temporal trends and prevalence in the use of clozapine polypharmacy at discharge among these patients from a major psychiatric hospital in southern Taiwan.

## 2. Methods

### 2.1. Ethics

This study was conducted at Kaohsiung Municipal Kai-Syuan Psychiatric Hospital, which is a public psychiatric center with the largest number of acute care psychiatric beds among all the psychiatric institutes in southern Taiwan. We hope that the large sample size would improve the representativeness of the study sample. The study received approval from Institutional Review Board of Kaohsiung Municipal Kai-Syuan Psychiatric Hospital (Approval No. KSPH-2023-01) and was conducted in compliance with both the Declaration of Helsinki (2013) and the Human Subjects Research Act in Taiwan, which governs national legislation regarding research involving human subjects. The ethics committees of the study hospital did not require informed consents, as all the collected data constituted routine clinical care and was delinked before analysis to guarantee anonymity.

### 2.2. Subjects

This study is a retrospective analysis in which patient data were extracted from the medical records and the electronic health information system at a major psychiatric hospital in southern Taiwan. Patients were eligible if they were discharged from the acute care wards to the community with a Diagnostic and Statistical Manual of Mental Disorders, 4th edition, Text Revision or Diagnostic and Statistical Manual of Mental Disorders, 5th edition diagnosis of schizophrenia or schizoaffective disorder^[21,22]^ and a discharge prescription of clozapine during the study period of January 1, 2006 through December 31, 2021. Diagnoses were made by board-certified psychiatrists, based on clinical observations and interviews during hospitalization, as well as previous medical records and information provided by the main caregiver. Patients who did not have complete prescription data available or were in a clinical trial during the study period were excluded. Patients were categorized into 2 groups: clozapine monotherapy (i.e., only clozapine, without any other antipsychotic) and clozapine polypharmacy (i.e., clozapine augmented with other antipsychotics). The following data were collected for analysis: age, sex, age of illness onset, number of previous hospitalizations, length of hospital stay, daily dose of clozapine, and daily dose of anticholinergics.^[23,24]^

### 2.3. Statistical analysis

The Pearson chi-squared test and the independent *t* test were used to compare demographic and clinical characteristics between clozapine monotherapy and clozapine polypharmacy. Multivariate logistic regression was used to identify factors associated with clozapine polypharmacy at discharge. Covariates included age, sex, onset of illness, length of hospital stay, number of previous hospitalizations, clozapine daily doses. The Cochran–Armitage trend test was used to evaluate whether significant time trends existed for rates of clozapine antipsychotic polypharmacy at discharge during the study period. Data were analyzed using SPSS version 27.0 for Windows (SPSS Inc, Chicago, IL) and SAS 9.4 software (SAS Institute Inc, Cary, NC). Statistical significance was defined as a *P*-value < .05.

## 3. Results

A total of 3271 patients with schizophrenia or schizoaffective disorder were discharged from the hospital with prescriptions for clozapine during the study period. Table 1 lists the demographic and clinical characteristics of these patients. Among the 3271 patients, 1886 (57.7%) were male, and 1385 (42.3%) were female. The average age of the cohort was 45.3 ± 11 years, the average age of onset was 25.0 ± 10.3 years, and the average length of hospital stay was 299.2 ± 510.7 days. The average number of previous hospitalizations was 6.2 ± 5.8 episodes. Regarding the antipsychotics prescribed, 1875 (57.3%) received only clozapine antipsychotic, while 1396 (42.7%) received clozapine antipsychotic polypharmacy. The average dosage of clozapine used was 229.4 ± 119.5 mg daily.

**Table 1:** Demographic and clinical characteristics of the study population (n = 3271).

	n	%
Sex		
Male	1886	57.7%
Female	1385	42.3%
Clozapine monotherapy	1875	57.3%
Clozapine polypharmacy*	1396	42.7%
	**Mean**	**SD**
Age (years)	45.3	11
Age of onset (years)	25.0	10.3
Length of hospital stay (days)	299.2	510.7
Number of previous hospitalizations	6.2	5.8
Clozapine dose (mg/d)	229.4	119.5

* Clozapine polypharmacy = clozapine augmented with other antipsychotics.

### 3.1. Clozapine monotherapy versus clozapine polypharmacy

Table 2 presents a comparison of demographic and clinical characteristics between patients who received clozapine monotherapy and those who received polypharmacy among the patients discharged on clozapine. Notably, the male sex was overrepresented among patients receiving clozapine polypharmacy, and these patients tended to be younger and experienced an earlier onset of illness compared to those receiving clozapine monotherapy. Additionally, an increased number of previous hospitalizations and shorter lengths of hospital stay were associated with higher rates of polypharmacy usage. Besides, patients on clozapine polypharmacy, in contrast to those on clozapine monotherapy, received lower doses of clozapine but higher doses of anticholinergic agents.

**Table 2:** A comparison between patients discharged on clozapine monotherapy and clozapine polypharmacy.

	Clozapine monotherapy (n = 1875, 57.3%)		Clozapine polypharmacy (n = 1396, 42.7%)		*P*
	n	%	n	%	
Sex					**.001** *
Male (n, %)	1035	55.2%	851	61.0%	
Female (n, %)	840	44.8%	545	39.0%	
	**Mean**	**SD** †	**Mean**	**SD** †	***P***
Age (years)	46	11.1	44.4	10.9	**<.001** ‡
Age of onset (years)	25.5	11	24.3	9.5	**.001** ‡
Number of previous hospitalizations	5.7	5.5	6.7	6.1	**<.001** ‡
Length of hospital stay (days)	337.3	551.5	247.9	445.2	**<.001** ‡
Clozapine dose (mg/d)	236.0	111.4	220.3	129	**<.001** ‡
Biperiden equivalent dose (mg/d)	1.0	2.5	2.1	3.0	**<.001** ‡

Bolded values are statistically significant.

* Pearson *χ*^2^ test.

† SD = standard deviation.

‡ Independent *t* test.

### 3.2. Factors associated with clozapine polypharmacy

Table 3 presents the factors most significantly associated with clozapine polypharmacy among all study participants (n = 3271), as determined by multivariate regression analysis. Sample size used in the present study to run logistic analysis is satisfactory. Older age (odds ratio, 0.992; *P* = .040), older age of onset (odds ratio, 0.988; *P* = .004), longer length of hospital stay (odds ratio, 0.9997; *P* = .001), higher clozapine dosage (odds ratio, 0.9989; *P* < .001) were found to be associated with clozapine monotherapy used. Male sex (odds ratio, 1.190; *P* = .018), increased numbers of previous hospitalizations (odds ratio, 1.021; *P* = .001) were found to be associated with increased clozapine polypharmacy.

**Table 3:** Factors associated with clozapine antipsychotic polypharmacy in multivariate regression analysis.

Factors	Odds ratio	95% CI*	*P*
Sex-male, n (%)	1.190	1.030–1.373	**.018**
Age (year), mean (SD)	0.992	0.9851–0.9996	**.040**
Age of onset (year), mean (SD)	0.988	0.9803–0.9962	**.004**
No. of previous hospitalizations, mean (SD)	1.021	1.0087–1.0343	**.001**
Length of hospital stay (days), mean (SD)	0.9997	0.9995–0.9999	**.001**
Clozapine daily dose (mg), mean (SD)	0.9989	0.9983–0.9995	**<.001**

Bolded values are statistically significant.

* CI = confidence interval.

### 3.3. Temporal trends in clozapine polypharmacy

Table 4 presents the percentage of patients discharged on clozapine polypharmacy between 2006 and 2021. The analysis of the Cochran–Armitage trend test indicated a significant increasing trend in the use of clozapine polypharmacy at discharge (*P* < .001), which rose from 22.4% in 2006 to 50% in 2021.

**Table 4:** Temporal trends in percentage of patients discharged on clozapine polypharmacy, 2006–2021.

Years	2006	2007	2008	2009	2010	2011	2012	2013	2014
Patients discharge on clozapine, n	134	142	157	189	195	169	225	214	227
Clozapine polypharmacy, n (%)	30 (22.4%)	51 (35.9%)	62 (39.5%)	72 (38.1%)	78 (40.0%)	73 (43.2%)	75 (33.3%)	77 (36.0%)	99 (43.6%)
Clozapine monotherapy, n (%)	104 (77.6%)	91 (64.1%)	95 (60.5%)	117 (61.9%)	117 (60.0%)	96 (56.8%)	150 (66.7%)	137 (64.0%)	128 (56.4%)
**Years**	**2015**	**2016**	**2017**	**2018**	**2019**	**2020**	**2021**		
Patients discharge on clozapine, n	176	205	210	225	246	271	286	*Z*	*P*
Clozapine polypharmacy, n (%)	88 (50.0%)	100 (48.8%)	91 (43.3%)	95 (42.2%)	115 (46.7%)	147 (54.2%)	143 (50.0%)	6.643	**<.001** *
Clozapine monotherapy, n (%)	88 (50.0%)	105 (51.2%)	119 (56.7%)	130 (57.8%)	131 (53.3%)	124 (45.8%)	143 (50.0%)		

* Cochran–Armitage trend test.

## 4. Discussion

To our knowledge, this is the most extensive study to date investigating factors and trend associated with antipsychotic polypharmacy in schizophrenia patients undergoing clozapine treatment. We identified factors associated with clozapine polypharmacy, including male sex, early onset of illness, younger age, a higher number of previous hospitalizations, shorter length of hospital stay, and lower clozapine dosage. We also demonstrated an increasing trend in the use of clozapine antipsychotic polypharmacy in southern Taiwan. The prevalence of clozapine antipsychotic polypharmacy significantly increased from 22.4% to 50% between 2006 and 2021, which roughly consistent with the prevalence reported by United Kingdom and Canada.^[19,20]^

We found that several demographic characteristics, such as sex and age, along with the course of the illness, were associated with the use of clozapine polypharmacy. It is not surprising that being male, younger in age, having an early onset of the illness, and experiencing frequent hospitalizations were predictive factors for polypharmacy usage. Two underlying reasons may explain this phenomenon. First, these individuals often have a more severe, treatment-resistant illness with a poorer prognosis. For example, males have been shown to have poor outcomes in schizophrenia clinical remission and recovery,^[25,26]^ and early onset of psychotic symptoms might be closely associated with resistance to antipsychotic treatment.^[27]^ Frequent hospitalizations are often indicative of recurrent episodes or a more severe course of the illness, which may require the use of different types of medications. These findings align with previous research on clozapine resistance. A shorter duration of illness, later onset of illness, and fewer hospitalizations exhibited a trend toward a significant association with a better response to clozapine.^[28]^ Some studies even suggested the use of other antipsychotic drugs, such as amisulpride or aripiprazole augmentation, in cases of clozapine-resistant schizophrenia.^[29–31]^ Second, male gender and young age are associated with a higher risk of aggression, which could influence prescription patterns. However, it is essential to note that there is no clear evidence that combination antipsychotic therapy is helpful in treating aggression or violence.^[32]^

Interestingly, in our study, we found that shorter hospital stays were more likely to be associated with clozapine polypharmacy, which contrasts with some prior research on polypharmacy. A case-control study suggested that brief administration of multiple antipsychotic medications was linked to increased drug exposure, adverse reactions, and longer hospitalization without evident clinical benefits.^[33]^ Another study indicated that the likelihood of being prescribed multiple medications was related to the length of stay in the facility.^[34]^ This discrepancy leads us to question whether the combination of clozapine with a second antipsychotic medication indeed expedites treatment effectiveness and is associated with shorter hospital stays.

In the realm of medication, our observations reveal 3 noteworthy findings. First, our observations suggest a correlation between lower clozapine dosage and clozapine antipsychotic polypharmacy. We suspect that this association arises when patients exhibit a lower tolerance to clozapine and experience a higher incidence of side effects, particularly those that are dose-dependent (such as obsessive-compulsive symptoms, heart rate variability, hyperinsulinemia, metabolic syndrome, and constipation).^[8]^ When clinicians find that the use of clozapine alone fails to achieve the desired therapeutic effect for psychosis, especially in cases where patients have poor tolerance and numerous dose-dependent side effects, they may choose to augment the treatment by combining clozapine with other antipsychotics to achieve the desired therapeutic outcome. Second, our research also revealed that the average dosage of clozapine used was 229.4 mg/d, which is below the target dose range of 300 to 450 mg/d.^[35]^ This is because Asians may require only half the clozapine dosage used in Western countries.^[36]^ Studies conducted in Taiwan have shown that, at the same dosage, the concentration of the drug in the blood is 30% to 50% higher in Asians than in Caucasians.^[37]^ Finally, our study revealed that patients with clozapine antipsychotic polypharmacy were associated with higher anticholinergic agent usage. Because clozapine is relatively less likely to induce extrapyramidal symptoms compared to other antipsychotic medications,^[38]^ when additional antipsychotics of a different class are introduced, the probability of experiencing extrapyramidal syndrome side effects increases. Therefore, the use of anticholinergic agents becomes necessary to manage these side effects.

We observe a clear trend indicating an increasing number of clinicians adopting the strategy of clozapine polypharmacy. This suggests that the proportion of clinicians achieving ideal therapeutic goals through the sole use of clozapine is decreasing, and the clinical landscape is becoming more complex. Additionally, psychiatrists, nursing staff, patients, and their families expect to see improvements in patients’ symptoms in a short time. The use of clozapine polypharmacy may be driven largely by the desire to achieve symptom reduction more rapidly. Clinicians are required to balance the pros and cons of medication dosage and types, patient characteristics, treatment efficacy, and side effects. In our study, we analyze the patient characteristics that are related to the eventual use of clozapine antipsychotic polypharmacy.

Nevertheless, certain limitations must be acknowledged when interpreting the findings of this study. Firstly, the study adopted a retrospective design, and the sample lacked randomization. Secondly, this study was conducted in a hospital setting, exclusively involving inpatients. Given that inpatients tend to be more severely or chronically ill and may exhibit treatment resistance, the study’s conclusions may not be readily applicable to outpatient populations. Third, this study site was one large public psychiatric hospital, the generalizability of the findings to general hospitals may be constrained. Fourth, in this study, it was found that the average length of hospitalization for this population was relatively long (an average of 299.2 days). Therefore, caution should be exercised when extrapolating the study results to all hospitalized patients. Fifth, we did not account for the potential impact of concurrent use of other psychotropic medications in the analysis, including antidepressants, mood stabilizers, and glutamatergic agents. Finally, this study did not collect data on certain crucial clinical characteristics, such as symptom severity, functional impairment, risk of suicide or violence, adverse events, and comorbidity. These data may also be related to the choice of medication.

## 5. Conclusion

In this study, we observed a growing trend among clinicians in adopting the clozapine antipsychotic polypharmacy prescribing pattern. We identified multiple factors associated with the utilization of clozapine polypharmacy, including male gender, younger, earlier age of onset, a greater number of previous hospitalizations, shorter length of hospital stay, and lower clozapine daily dose. Although augmentation with a second antipsychotic may offer modest benefits for patients who do not respond fully to clozapine, it is crucial to carefully weigh the risks and side effects associated with antipsychotic polypharmacy. The use of clozapine polypharmacy remains a topic of debate, and further research is required to thoroughly elucidate its efficacy, safety, and overall risk/benefit ratio. This will contribute to providing clinicians with more detailed insights and recommendations for prescribing strategies.

## Author contributions

**Conceptualization:** Yu-Ju Shih.

**Data curation:** Yu-Ju Shih, Ching-Hua Lin.

**Formal analysis:** Yu-Ju Shih, Li-Shiu Chou.

**Investigation:** Yu-Ju Shih, Ching-Hua Lin.

**Methodology:** Yu-Ju Shih, Ching-Hua Lin, Li-Shiu Chou.

**Software:** Yu-Ju Shih.

**Validation:** Ching-Hua Lin.

**Writing—original draft:** Yu-Ju Shih.

**Writing—review & editing:** Yu-Ju Shih, Ching-Hua Lin, Li-Shiu Chou.
